# Imprisoned and imperiled: access to HIV and TB prevention and treatment, and denial of human rights, in Zambian prisons

**DOI:** 10.1186/1758-2652-14-8

**Published:** 2011-02-11

**Authors:** Katherine W Todrys, Joseph J Amon, Godfrey Malembeka, Michaela Clayton

**Affiliations:** 1 Human Rights Watch, London, UK; 2 Human Rights Watch, New York, USA; 3 Prisons Care and Counselling Association, Lusaka, Zambia; 4 AIDS and Rights Alliance for Southern Africa, Windhoek, Namibia

## Abstract

**Background:**

Although HIV and tuberculosis (TB) prevalence are high in prisons throughout sub-Saharan Africa, little research has been conducted on factors related to prevention, testing and treatment services.

**Methods:**

To better understand the relationship between prison conditions, the criminal justice system, and HIV and TB in Zambian prisons, we conducted a mixed-method study, including: facility assessments and in-depth interviews with 246 prisoners and 30 prison officers at six Zambian prisons; a review of Zambian legislation and policy governing prisons and the criminal justice system; and 46 key informant interviews with government and non-governmental organization officials and representatives of international agencies and donors.

**Results:**

The facility assessments, in-depth interviews and key informant interviews found serious barriers to HIV and TB prevention and treatment, and extended pre-trial detention that contributed to overcrowded conditions. Disparities both between prisons and among different categories of prisoners within prisons were noted, with juveniles, women, pre-trial detainees and immigration detainees significantly less likely to access health services.

**Conclusions:**

Current conditions and the lack of available medical care in Zambia's prisons violate human rights protections and threaten prisoners' health. In order to protect the health of prisoners, prison-based health services, linkages to community-based health care, general prison conditions and failures of the criminal justice system that exacerbate overcrowding must be immediately improved. International donors should work with the Zambian government to support prison and justice system reform and ensure that their provision of funding in such areas as health services respect human rights standards, including non-discrimination. Human rights protections against torture and cruel, inhuman or degrading treatment, and criminal justice system rights, are essential to curbing the spread of HIV and TB in Zambian prisons, and to achieving broader goals to reduce HIV and TB in Zambia.

## Background

Current conditions in prisons in many African countries are life threatening. HIV prevalence among prisoners in sub-Saharan African prisons has been estimated at two to 50 times the prevalence in non-prison populations [1], and tuberculosis (TB) prevalence at six to 30 times that of national rates [2,3]. Overcrowding, caused by lack of investment and poorly functioning criminal justice systems, is endemic [1,4], and is a contributing factor, along with violence, food insecurity and minimal access to health care or prevention, to HIV and TB transmission, morbidity and mortality [1,5-10].

In the past decade, Zambia has dramatically expanded its national response to HIV and TB. In 2004, the Zambian government introduced free antiretroviral therapy (ART), and in June 2005, it declared all ART-related services free [11]. Between 2004 and 2007, the number of people on ART increased from 20,000 to 151,000 [11].

However, HIV prevalence among Zambian adults remains high, with an estimated 15% adult prevalence [11], and a total of 1.1 million HIV-infected individuals [11]. Among prisoners, research from 1999, the most recent available, found 27% of male and 33% of female prisoners to be infected [12]. National TB prevalence, among the highest in the world, was estimated to be 0.4% in 2007 [13]; among prison populations, prevalence was estimated to be between 15% and 20% in 2001 [14]. The Zambia Prisons Service (ZPS) has estimated that between 1995 and 2000, 2397 inmates and 263 prison staff died from AIDS-related illnesses, including TB [15].

Despite increasing attention among international agencies and donors to the problem of HIV and TB in African prisons [1], few resources have been devoted to improving conditions in prisons generally, or to addressing HIV and TB prevention, treatment or care specifically. While donors have generously supported health initiatives in Zambia over the past decade, little funding has gone to government or non-governmental organization (NGO)-based prison health initiatives.

In 2009, the United States contributed more than US$262 million, and the Global Fund to Fight AIDS, TB and Malaria contributed more than US$137 million, to HIV programmes in Zambia [16]. Yet, in 2008, when the National HIV/AIDS/STI/TB Council analyzed donor spending for HIV/AIDS programmes in Zambia they found that US$0 was spent on HIV programmes for prisoners in 2005 and only US$76,300 was spent in 2006 [17]. According to Zambian prison officials, the entire health budget of the ZPS (excluding salaries) was US$0 in 2009 and US$42,210 in 2010.

Zambian prisons were at more than 300% of capacity in April 2010: built to accommodate 5500 prisoners before Zambian independence in 1964 [18], the country's prisons housed 16,666 in 2010 [Chisela Chileshe, medical director, ZPS]. To better understand the relationship between prison conditions, criminal justice rights, and HIV and TB prevention, treatment and care in Zambia, we conducted facility assessments and interviews with prisoners and prison officers in six prisons, and interviews with government and NGO key informants. We also reviewed Zambian laws and policies and international human rights laws and standards related to prison, HIV and TB.

## Methods

Zambia has a total of 86 prisons. Thirty-three are "open-air," or farm prisons, and 53 are "standard" prisons. Juvenile and female prisoners are incarcerated in facilities throughout the country as well as in one dedicated juvenile prison and another exclusively female prison.

For the present investigation, a mixed-method study was designed, which included: 1) a brief prisoner survey and longer, semi-structured in-depth interviews; 2) semi-structured interviews with prison officers; 3) facility assessments of the prisons in which inmates and prison officers were interviewed; 4) key informant interviews with the Zambian government and NGO officials; and 5) an analysis of the national laws and policies governing the Zambian prison and criminal justice systems.

This methodology was chosen in order to develop a comprehensive understanding of the conditions faced by prisoners, primarily through prisoners' self-reporting, but also through information provided by prison officials and key informants, and through information on prison and justice system laws and policies. Prisoners were asked to complete both a survey and an in-depth interview to provide a way of systematically presenting key indicators, as well as of allowing more thorough documentation of conditions and nuanced understanding of the interrelation of key variables.

Prisoner and prison officer interviews were conducted in six prisons throughout the central corridor of Zambia, including: three urban prisons, Lusaka Central Prison (Lusaka Province), Mukobeko Maximum Security Prison (Central Province) and Kamfinsa State Prison (Copperbelt Province); one rural district prison, Mumbwa Prison (Central Province); and two peri-urban prisons, Mwembeshi Commercial Open Air Farm Prison (Central Province) and Choma State Prison (Southern Province). Prisons were selected based on their diverse location, size and security level, and because of ongoing participation with an HIV peer-education programme conducted by one organization participating in the research, the Prisons Care and Counselling Association (PRISCCA).

In each prison visited, researchers requested from the officer in charge a private location to conduct interviews with a cross-section of prisoners held in that facility, including female prisoners, immigration detainees, juveniles (classified under Zambian law as inmates aged eight to 18) and unconvicted ("remandee") detainees. Priority was given to the inclusion of prisoners from each category, rather than proportional representation. Officers identified prisoners who were then provided by researchers with a verbal explanation of the survey (in English or French, and translated into Bemba, Nyanja or Tonga if necessary), asked if they were willing to participate, and assured of anonymity. Individuals were assured that they could decline to participate, end the interview at any time, or decline to answer any specific questions without negative consequence. The names of all prisoners who participated in this study have been changed to protect their anonymity and security.

Each interview took approximately 45 minutes and was conducted in English or French by researchers from one of three organizations - Human Rights Watch, PRISCCA, or the AIDS and Rights Alliance for Southern Africa (ARASA) - or in Bemba, Nyanja or Tonga, with translation into English provided by members of PRISCCA. Interviewers used a brief verbal questionnaire to gather information on each prisoner's incarceration history, medical care, and experience of HIV/AIDS and TB testing and treatment. Researchers then probed responses and asked further questions regarding prison conditions, discipline and HIV/TB risk behaviour in open-ended, in-depth interviews. All interviews were conducted outside of the hearing of prison officers and other prisoners, in a private setting. The number of interviews at each prison was limited by the Zambia Prisons Service, which allowed access to each prison for a fixed period of time.

At each facility visited, researchers requested interviewing the officer in charge, deputy officer in charge, medical officer and female officer in charge; additional officers were invited to participate if sufficient time allowed. Prison officers were provided with an explanation of the purpose of the study and how the information obtained would be used; they were given the opportunity to decline the interview or to end the interview at any time. Prison officer interviews focused on HIV and tuberculosis testing and treatment availability in the prison, healthcare delivery, deaths in custody, prison administration, prisoner discipline and treatment, and prison officers' working conditions.

Quantitative interview data from prisoners were entered using the Statistical Package for the Social Sciences (version release 11.0.1, SPSS Inc., Chicago, Illinois), and analyzed for frequency of key variables, stratified by prison and prisoner characteristics. Chi square tests were used to compare differences in categorical variables.

Qualitative prisoner data were transcribed and hand-coded and the authors conducted a content analysis to identify key themes corresponding to the interview guide, as well as emergent topics. In the first analysis of the data, an initial set of codes was generated to capture key constructs. Subsequent analyses were undertaken to examine the consistency of reports across themes and examine negative evidence [19].

The facility assessments examined the condition of prison facilities, and the proximity and availability of medical care. Each assessment included a visit to prisoner cells, any medical facilities, prison common areas, and bathroom/shower facilities. Visits to punishment and medical isolation cells were also requested at all facilities, and granted at Mumbwa Prison (punishment cells) and Lusaka Central Prison (isolation cells).

Interviews with key informants from government and national and international NGOs were also conducted, prior to and following prison-based interviews, to identify salient issues and probe specific findings raised in the research. Finally, national legislation and policy governing the administration of the prison and criminal justice systems were reviewed.

Information from facility assessments, interviews with government and NGO officials, and legal and policy reviews were organized by theme and used to inform the analysis of prisoner testimony and the development of key recommendations, as part of a report published elsewhere [20].

Human Rights Watch does not generally identify its work as "research", defined as seeking to develop "generalizable knowledge" [21]. Rather, our investigations aim to document and respond to specific human rights abuses, monitor human rights conditions, and assess human rights protections in specific settings. Each of these purposes is consistent with what has been defined as "public health non-research" [22] or practice [21]. However, because public health non-research and practice also raise ethical and human participant protection issues, all investigations conducted by Human Rights Watch are subject to rigorous internal review, and external ethics and subject-area experts are consulted when investigations involve particularly difficult settings, populations or issues.

The present study's methods, and human participant protections associated with the research, were reviewed and approved by PRISCCA, ARASA and Human Rights Watch prior to undertaking this study, and all interviewers were trained in human participant protection and information security. The study protocol, including detailed information on security measures to be taken to protect interviewers, key informants and individuals - particularly the prisoners and prison officers who were witnesses to and victims of human rights violations - was reviewed and approved by staff in Human Rights Watch's Health and Human Rights, Africa, Women's Rights, Children's Rights, and Lesbian, Gay, Bisexual, and Transgender Rights divisions. It was also reviewed by the legal and policy department, and by the organization's programme director.

In addition, a post-research memorandum was written that documented potential risks to participants and ethical issues that arose during the research and the steps taken in response. Further, publications of the results of the investigation were reviewed and approved by the divisions, just named, to ensure that informants were not identifiable and human participant protections were respected. Anonymized prisoner data, and non-anonymized prison officer and key informant interview data from the study are stored securely with Human Rights Watch.

In addition, in July 2009, PRISCCA sought permission from the Zambian Ministry of Home Affairs and Ministry of Foreign Affairs for individuals from all three organizations to enter Zambian prisons to conduct research. In September 2009, both ministries granted permission.

## Results

Between September 2009 and February 2010, 246 prisoners in six prisons were asked to participate in the study, and all consented. Fourteen prisoners were asked only to complete in-depth interviews, and 232 completed both the quantitative survey and an in-depth interview (Table 1). In addition, 31 prison officers and 18 Zambian government officials from relevant ministries were approached for interviews; one prison officer declined. Twenty-eight representatives from local and international NGOs, and donor governments and agencies were also interviewed.

**Table 1:** Self-reported characteristics of prisoners completing quantitative survey on healthcare and incarceration status at six Zambian Prisons, September 2009-February 2010

	Lusaka Central (n = 62)		Mukobeko (n = 51)		Kamfinsa (n = 39)		Mumbwa (n = 26)		Mwembeshi (n = 27)		Choma (n = 27)		Overall (six prisons) (n = 232)	
By sex														

Female	37% (23)		N/A		28% (11)		4% (1)		N/A		26% (7)		18% (42)	

Male	63% (39)		100% (51)		72% (28)		96% (25)		100% (27)		74% (20)		82% (190)	

By legal classification	male	female	m	f	m	f	m	f	m	f	m	f	m	f

Adult convicts (19 years and older)	46% (18)	48% (11)	80% (41)	N/A	64% (18)	55% (6)	68% (17)	0% (0)	100% (27)	N/A	65% (13)	43% (3)	71% (134)	48% (20)

Adult remandees (19 years and older)	28% (11)	30% (7)	8% (4)	N/A	11% (3)	36% (4)	26% (7)	100% (1)	0% (0)	N/A	0% (0)	29% (2)	13% (25)	33% (14)

Adult immigration detainees (19 years and older)	13% (5)	13% (3)	0% (0)	N/A	21% (6)	9% (1)	0% (0)	0% (0)	0% (0)	N/A	5% (1)	0% (0)	6% (12)	10% (4)

Juveniles (8-18 years)	13% (5)	9% (2)	12% (6)	N/A	4% (1)	0% (0)	4% (1)	0% (0)	0% (0)	N/A	30% (6)	29% (2)	10% (19)	10% (4)

### General access to health care

In 2010, the Zambia Prisons Service employed 14 trained health staff, including one physician, for a prison population of 16,666. Only 15 of Zambia's 86 prisons included health clinics or sick bays [Chisela Chileshe, medical director, ZPS]. Even when clinics do exist, many have little capacity beyond distributing paracetemol [23] [facility assessments of Lusaka Central Prison, Mukobeko Maximum Security Prison, Kamfinsa State Prison, Mumbwa Prison, Mwembeshi Commercial Open Air Farm Prison and Choma State Prison]. According to ZPS staff and prison officers, in prisons without a medical clinic - and for prisoners with more serious medical conditions requiring advanced care - access to care is frequently controlled by medically unqualified and untrained prison officers who evaluate and determine if medical visits to community health facilities are necessary.

Prisoners and prison officials at each of the six prisons visited also blamed the lack of sufficient prison staff, transportation and fuel, as well as security fears, for lengthy delays in the transfer of sick prisoners to medical care outside of the prisons, in some cases for days or weeks after they fall ill.

At prisons with associated farm facilities (Mumbwa Prison and Mwembeshi Commercial Open Air Farm Prison), inmates consistently reported that the requirement to work long hours frequently prevented them from accessing necessary medical care [inmates Gabriel and Febian at Mumbwa Prison; inmates Rabun and Jacob at Mwembeshi Commercial Open Air Farm Prison]. As the inmate Jacob reported, "It is not possible here to go to the doctor. At the moment we wake up, we go to the field, then we go to a different field. Even if you complain [that you are sick], the officers tell you that you still have to go."

### Tuberculosis screening and care

Wide variation in rates of TB testing since incarceration was seen among prisoners in different facilities and between inmate groups within each prison. TB testing rates were based upon self-reports of prisoners, and defined broadly to include clinical examination, sputum analysis and chest X-ray. Testing was higher in larger, urban facilities, namely, Lusaka Central (18%), Mukobeko Maximum Security (49%), and Kamfinsa State (32%), and lower in smaller, rural facilities, namely, Mumbwa (4%), Mwembeshi Commercial Open Air Farm (0%), and Choma State (11%) (p < 0.0001) (Table 2). Adult female prisoners (11%) were less likely to be tested than adult male prisoners (28%) (p < 0.05), juveniles (4%) were less likely to be tested than adults (25%) (p < 0.05), and remandees (12%) and immigration detainees (6%) were less likely to have been tested for TB than convicted prisoners (28%) (p = 0.05 for remandees; p < 0.01 for immigration detainees compared with convicted prisoners).

**Table 2:** TB testing by prisoner type: prisoners who self-reported having been tested for TB while incarcerated at six Zambian prisons, September 2009-February 2010

	Lusaka Central	Mukobeko	Kamfinsa	Mumbwa	Mwembeshi	Choma	Overall (six prisons)
Overall (%)	18	49	32	4	0	11	23

By age (%)							

Adults (19 years and older)	20	53	32	4	0	16	25

Males	27	53	39	4	0	21	28

Females	10	N/A	18	0	N/A	0	11

Juveniles (8-18 years)	0	17	0	0	N/A	0	4

By classification (%)							

Convicts	16	56	50	6	0	19	28

Remandees	23	20	0	0	N/A	0	12

Immigration detainees	11	N/A	0	N/A	N/A	0	6

Prisoners and prison officers reported lengthy delays between experiencing symptoms of TB and having access to diagnostic tests; the medical officer at Mukobeko Maximum Security Prison told researchers that TB was often the last cause of illness tested for when an inmate presented with coughing, and treatment for upper respiratory infections was exhausted prior to testing for TB. Prison medical authorities said that routine TB screening was not conducted [Chisela Chileshe, medical director, ZPS], and TB screening of HIV-infected prisoners was uneven: 94% (16 out of 17) of inmates at Mukobeko Maximum Security Prison who self-identified as HIV infected had received a TB test, while none of the 10 self-identified HIV-infected inmates at Mwembeshi Commercial Open Air Farm Prison had been tested.

While an initial course of treatment is provided for all prisoners diagnosed with TB [Chisela Chileshe, medical director, ZPS; Nathan Kapata, director of the national tuberculosis programme, Ministry of Health; Helen Ayles, project coordinator, ZAMBART], we found no testing and treatment for drug resistance, even for inmates who had previously been treated for TB and whose symptoms persisted or who appeared to be treatment failures [Gabriel, inmate, Mumbwa Prison; nurse, Lusaka Central Prison]. Healthcare staff often do not know what medications prisoners have previously taken for TB [nurse, Lusaka Central Prison]. However, drug resistance testing and treatment in Zambian medical facilities are also inconsistent and not widely available [Helen Ayles, project coordinator, ZAMBART].

Standard isolation of TB infectious prisoners was rare, and practiced, according to prison medical authorities, in only "two or three" of the country's 86 prisons. Even where isolation exists, only patients diagnosed with TB are isolated; inmates with suspected TB based on their symptoms typically remain in the general population until diagnosis [Chisela Chileshe, medical director, ZPS].

On the days of researchers' visits, interviews with officers in charge indicated that Lusaka Central Prison, Mukobeko Maximum Security Prison, Kamfinsa State Prison and Choma State Prison had some form of facility they considered TB isolation. Observation of the 10-by-8-metre TB isolation cell during the facility assessment at Lusaka Central Prison in February 2010 found it to be crowded with 57 inmates, dirty, dark and with little ventilation. At Mukobeko Maximum Security Prison, the medical officer informed researchers that TB isolation facilities were improvised and conditions "pathetic".

According to ZPS medical staff, prison officers and prisoners, healthy inmates, TB- and non-TB-infected patients were routinely mixed in isolation cells. At Mukobeko Maximum Security Prison, healthy juvenile inmates were put in the TB isolation cell to protect them from more violent, overcrowded adult cells [inmates Phiri and Isaac at Mukobeko Maximum Security Prison]. At Lusaka Central Prison, the facility assessment found that among the 57 inmates in the "isolation" cell, 34 or fewer were receiving TB treatment.

Both prisoners and prison officers reported that prisoners commonly remained in isolation after completing TB treatment to avoid returning to even more overcrowded general population cells. As Kachinga, an inmate at Lusaka Central Prison who had completed TB treatment, noted, "I was tested for TB and put into the [isolation] cell. I tested positive. I finished my course of treatment, tested again, and was negative. I am still in the [TB isolation] cell. I would love to move out, to give room to other patients coming in, but the other cells are congested. It's my choice to stay."

### HIV/AIDS prevention, testing and treatment

Prisoners reported having been tested for HIV since incarceration more frequently than having been tested for TB, but HIV testing was also subject to inter- and intra-prison variability. Larger facilities had higher self-reported HIV testing rates among prisoners interviewed, ranging from 54% at Lusaka Central to 86% at Mukobeko Maximum Security; smaller facilities' HIV testing rates ranged from 23% at Mumbwa Prison to 48% at Mwembeshi Commercial Open Air Farm Prison (p < 0.0001) (Table 3).

**Table 3:** HIV testing by prisoner type: prisoners who self-reported having been tested for HIV while incarcerated at six Zambian prisons, September 2009-February 2010

	Lusaka Central	Mukobeko	Kamfinsa	Mumbwa	Mwembeshi	Choma	Overall (six prisons)
Overall (%)	54	86	72	23	48	33	57

By age (%)							

Adults (19 years and older)	54	89	74	20	48	42	59

Males	68	89	71	21	48	50	62

Females	33	N/A	82	0	N/A	20	45

Juveniles (8-18 years)	57	67	0	100	N/A	13	44

By classification (%)							

Convicts	53	90	92	33	48	44	65

Remandees	62	70	57	0	N/A	20	46

Immigration detainees	38	N/A	0	N/A	N/A	0	21

Voluntary prison-based HIV testing is conducted in only six of the country's 86 prisons [Chisela Chileshe, medical director, ZPS]. Of the six prisons visited, facility assessments revealed that only three (Mukobeko Maximum Security Prison, Lusaka Central Prison and Mwembeshi Commercial Open Air Farm Prison) participated in the testing programme run by the Go Centre/CHRESO Ministries programme. In other prisons, diagnostic HIV testing may be conducted if it is indicated and a prisoner is able to access care.

As with TB testing, certain categories of inmates, including women, juveniles, remandees and immigration detainees, reported being tested for HIV less frequently than their adult, male convict counterparts. Adult female prisoners (45%) were less likely to be tested than adult male prisoners (62%) (p < 0.05), juveniles (44%) were less likely to be tested than adults (59%) (p = ns), and remandees (46%) and immigration detainees (21%) were less likely to have been tested for HIV than convicted prisoners (65%) (p < 0.05 for remandees; p < 0.001 for immigration detainees compared with convicted prisoners).

For inmates who had tested positive for HIV, ART was often available at the prison referral hospital or through the Go Centre/CHRESO at the six prison facilities it serves. Of the prisoners interviewed who reported having tested positive for HIV, 60% had been started on some form of treatment, including ART. Prisoners at larger prisons were more likely to have been started on treatment than their counterparts at smaller, rural prisons [20]. Cotrimoxazole prophylaxis, recommended for all individuals testing positive for HIV in order to prevent opportunistic infections, is almost entirely unavailable at all prisons, and only one prisoner interviewed reported having been started on it after testing positive for HIV. By contrast, cotrimoxazole is generally available at all Ministry of Health ART clinics [Steward Reid, CIDRZ].

Among inmates on ART interviewed (n = 18), more than half (n = 10) had missed doses. Reasons for missing doses included lack of food (n = 7), lack of transportation to clinics (n = 3) and unavailability of treatment (n = 2). Willard, 25, an HIV-positive inmate at Mukobeko Maximum Security Prison, reported, "They used to give extra food for taking medications but no extra food now. It is hard to take these very strong drugs without enough food." Both prisoners and prison officers routinely noted the health effects of lack of nutritional supplements for HIV and TB patients.

More than 40 inmates reported that sexual activity between male inmates was common, including rape, consensual sex between adults, and relationships where sex was traded by the most vulnerable, especially juveniles, in exchange for protection, food, soap and other basic necessities not provided by the prison. Several prison officers denied the occurrence of sexual activity [officers in charge at Mukobeko Maximum Security Prison, Kamfinsa State Prison Mumbwa Prison and Choma State Prison]; though others admitted that it occurs [deputy officer in charge, Mukobeko Maximum Security Prison; prison officer, Mukobeko Maximum Security Prison; officer in charge, Lusaka Central Prison; deputy officer in charge, Mumbwa Prison].

Zambian policy acknowledges, "Prison confinement can increase vulnerability to HIV due to frequent unprotected sex in the form of rape, non-availability and non-use of condoms, as well as high prevalence of STIs" [24], and prison officials acknowledged their obligation to ensure that HIV prevention methods available to Zambians outside of prisons are equally available to those imprisoned [Chisela Chileshe, medical director, ZPS]. Further, noting that "[p]revention is better than cure", the Zambia Prisons Service has set for itself the goal of ensuring "the implementation of a comprehensive HIV prevention package" [15].

However, facility assessments found a complete unavailability of condoms in all prisons visited. Official and unofficial punishment for engaging in sexual activity is enforced, in some cases brutally [officers in charge at Mukobeko Maximum Security Prison, Kamfinsa State Prison, Mumbwa Prison, Choma State Prison, Lusaka Central Prison and Mwembeshi Commercial Open Air Farm Prison; inmates Chiluba, Albert and Moses at Lusaka Central Prison; inmates Keith and Mumba at Mukobeko Maximum Security Prison].

### Overcrowding and abuse

At Lusaka Central Prison and Mukobeko Maximum Security Prison, facility tours and interviews with inmates found overcrowding so severe that inmates sometimes had to sleep seated or in shifts; at other prisons, inmates reported that they slept on their sides, up to five on a mattress, unable to turn over [inmates Arthur and Gideon at Mwembeshi Commercial Open Air Farm Prison; Noah, inmate, Mumbwa Prison]. Albert, 30 years old, an unconvicted inmate at Lusaka Central Prison, reported, "We are not able to lie down. We have to spend the entire night sitting up. We sit back against the wall with others in front of us. Some manage to sleep, but the arrangement is very difficult. We are arranged like firewood." Facility assessments at all six prisons found that ventilation in cells was limited to small windows, and prisoners were frequently confined to their cells for 14 hours each night.

Inmates reported being subjected to corporal punishment and "penal block" isolation practices, where prisoners are stripped naked and left in a small, windowless cell while officers pour water onto the floor to reach ankle or mid-calf height. There is no toilet in the cell, so inmates must stand in water containing their own excrement [facility assessment of Mumbwa Prison; Elijah, inmate, Mukobeko Maximum Security Prison; Joshua, inmate, Lusaka Central Prison; Andrew, inmate, Mumbwa Prison; Ngwila, inmate, Choma State Prison]. Prisoners also reported and some prison officers confirmed that certain inmates, appointed as "cell captains" by officers, are invested with disciplinary authority [officers in charge at Mumbwa Prison and Lusaka Central Prison; Frederick Chilukutu, deputy commissioner of prisons, ZPS] and judge fellow inmates and mete out punishments, including beatings, through night-time courts in their cells.

According to both inmates and prison officials, drinking water in prisons is scarce and sometimes unpotable [offender management officer, Mwembeshi Commercial Open Air Farm Prison; Douglas, inmate, Mukobeko Maximum Security Prison; Esnart, inmate, Lusaka Central Prison; Bianca, inmate, Kamfinsa State Prison; Harrison, inmate, Mumbwa Prison]; hygiene is poor, and soap and razors are not provided by the government [Catherine, inmate, Lusaka Central Prison; HIV/AIDS coordinator, Lusaka Central Prison]. Food is inadequate, and prison officers reported malnutrition-related illnesses and deaths [medical officer, Mukobeko Maximum Security Prison; Chisela Chileshe, medical director, ZPS; deputy officer in charge, Mukobeko Maximum Security Prison; medical officer, Choma State Prison].

### Criminal justice system failures

A wide range of problems were identified by inmates and key informants in relation to the criminal justice system and the realization of the rights of individuals accused or convicted of crimes. Police commonly arrest and hold alleged co-conspirators or family members when their primary targets cannot be found [18] [inmates Catherine, Angela and Susan at Lusaka Central Prison]. Such wholesale arrests may, in some cases, be sanctioned by Zambian law [25,26]. Significant delays occur before detainees are presented to a magistrate or judge, before their case is adjudicated by the court, and before any appeals are heard.

Ninety-seven percent of the prisoners interviewed had not seen a magistrate or judge within 24 hours of arrest (Table 4), even though such review is required under Zambian law [25]. On average, adult male detainees had spent four months in detention prior to seeing a judge or magistrate for the first time; adult female detainees had spent an average of one month in detention (Table 4). The average time at some prisons was even longer: at Kamfinsa State Prison, male detainees had averaged nine months between arrest and first appearance before a judge; at Mukobeko Maximum Security Prison, male detainees had averaged five months. Felix, an inmate at Mukobeko Maximum Security Prison, reported that his first appearance before a magistrate or judge had been three years and seven months after arrest. As Rodgers, a remandee at Lusaka Central Prison, concluded, "Justice delayed is justice denied. It is better even to be found guilty. When you come out, you've spent 10 years in prison. Remandees are kept here a long time. I have [been detained] four years now, but my case is not disposed of. There is no justice."

**Table 4:** Prisoners' self-reported access to the criminal justice system at six Zambian prisons, September 2009-February 2010

Prisoner category	% of prisoners who reported that they saw a judge within 24 hours of arrest	Length of time (months) between arrest and first appearance before a judge (average)	% of prisoners who reported being continuously detained from arrest (not receiving police bond or bail)	Time in detention (months) reported by remandees (median (range))
Overall	3	3	86	10 (0-67)

Adults (19 years and older)	2	3	85	36 (1-67)

Males	2	4	88	1 (0-28)

Females	3	1	75	5 (0-43)

Juveniles (8-18 years)	5	2	93	7 (0-67)

Among the prisoners interviewed, 95% of juveniles, 88% of adult males and 75% of adult females had been continuously detained from the time of their arrest, without having been released on police bond or bail (Table 4).

Following their initial appearance in front of a magistrate or judge, prisoners also reported waiting long periods before being tried, a phenomenon confirmed by additional human rights monitors [18]. Two inmates reported having been held on remand for six years [inmates Elijah and Mumba at Mukobeko Maximum Security Prison], and one reported having been held for 10 years before conviction [Arthur, inmate, Lusaka Central Prison]. Among current remandees interviewed, the median time held was 36 months for adult males, with a minimum of one month and a maximum of 67 months. For remanded juveniles, the median was five months, with a range from zero to a high of 43 months; for adult females, the median was one month, with a range from zero to 28 months (Table 4). "The long stay of prisoners without trial," Chishala, an inmate at Mukobeko Maximum Security Prison, said, "is unbearable."

For many of those who have been convicted, non-custodial sentencing options are unavailable. According to government and NGO officials, a 2000 law providing for non-custodial sentences has had minimal impact because of the lack of personnel to supervise those on community service orders [Frederick Chilukutu, deputy commissioner of prisons, ZPS; Chipo Mushota Nkhata, HIV/AIDS and human rights programmes officer, Human Rights Commission]. While community service orders were placed under the authority of the ZPS, no additional resources or staff to implement these orders were provided [27].

Parole has recently become available to inmates. However, only inmates with longer sentences - those who have been found guilty of more serious crimes - are eligible for parole, whereas inmates with more minor sentences are ineligible [officer in charge, Mwembeshi Commercial Open Air Farm Prison; Frederick Chilukutu, deputy commissioner of prisons, ZPS]. Additionally, the appeal process suffers from delays that can last for years [Arthur, inmate, Lusaka Central Prison; inmates Howard, Paul and Emmanuel at Mukobeko Maximum Security Prison]. As one "condemned" prisoner, Paul, at Mukobeko Maximum Security Prison, said, "My appeal has taken since 2005. I can no longer afford a lawyer to move it through the system. We are 235 in the condemned section. Only 40 have had their appeals heard. One hundred and eighty are still waiting, some for over 10 years."

## Discussion

In 2006, the United Nations General Assembly, on behalf of 192 country members, pledged its commitment to universal access to HIV prevention, treatment and care [28]. In line with this goal, Zambia has outlined an aggressive approach to addressing HIV and TB [29]. Foreign donors and multilateral agencies have provided significant health funding to Zambia to pursue these goals, with the United States Government and the Global Fund to Fight AIDS, Tuberculosis and Malaria providing the Zambian government with a combined US$1.2 billion in funds toward HIV between 2003 and 2009 [16]. Yet Zambian prisons are desperately and chronically underfunded, and prisoners face inhuman conditions, human rights abuses, and woefully inadequate access to HIV and TB prevention, treatment or care.

High vulnerability to HIV and TB among prisoners is widely acknowledged by international health agencies. In 1993, the World Health Organization (WHO) recognized the need for "vigorous efforts" to detect TB cases through entry and regular screenings in prisons, and the need for effective treatment programmes and continuity of treatment upon transfer or release [30]. In Zambian prisons, however, routine TB screening is not occurring, TB is often the last cause of illness suspected, and TB isolation is, even in the words of prison medical staff, "pathetic".

While the WHO has noted that appropriate treatment for drug-resistant TB includes the use of second-line drugs, with individual case management including a history of drug use in the country and the individual [31], such procedures are unheard of in Zambian prisons. Treatment for drug resistance simply does not exist, and treatment for drug-susceptible TB is sub-standard. TB isolation facilities are likely a key site of TB infection, including of multi-drug resistant strains, as individuals are crowded into dark, unventilated "isolation" cells, and stay in them even after the completion of therapy.

Although greater progress has been made in regard to HIV testing and treatment, stark inequalities between and within prisons persist, and the most vulnerable prisoners are those least likely to be tested. In the past several years, access to ART has increased exponentially in Zambia and a majority of HIV-infected Zambians who need treatment now have access to it. However, in Zambian prisons, access to both testing and treatment depends upon the prisoner's age, sex and legal classification, and on the prison to which the prisoner is assigned.

These disparities with respect to TB and HIV testing may be attributable to a number of factors. The disparity in testing between convicted and unconvicted detainees may be a result of officers' security fears in allowing remandees to leave the prison confines to go to a health clinic, the only place where TB testing and treatment are available, and a dispute between the prison and police authorities over responsibility for remandees' security. The disparity between convicted prisoners and immigration detainees could be attributable to discrimination experienced by immigration detainees in accessing care, and the fact that immigration detainees had, on average, spent less time in detention than convicted and remanded detainees.

The difference between adult male prisoners' access to TB and HIV testing and that of their female or juvenile counterparts is possibly attributable to a combination of factors: women and juveniles had, on average, been detained and incarcerated in their current facility for a shorter time than their male counterparts; juveniles (but not women) reported experiencing fewer health problems during incarceration and thus were probably less likely to visit health facilities; and female inmates were less educated than male inmates and perhaps less aware of and able to request testing.

Beyond these explanations, however, is a question of political will and respect for human rights. Under the international human rights treaties to which Zambia is a party, prisoners retain their human rights and fundamental freedoms, except for such restrictions on their rights required by the fact of incarceration; the conditions of detention should not aggravate the suffering inherent in imprisonment [32-34]. This principle is not dependent on the material resources available to the national government in question [33].

Also absolute is the obligation of the Zambian government to protect prisoners from torture. The International Covenant on Civil and Political Rights and the Convention Against Torture, to which Zambia is a party, prohibit torture and cruel, inhuman or degrading treatment or punishment without exception or derogation [35,36]. States have an obligation to ensure medical care for prisoners at least equivalent to that available to the general population [33-38], a commitment acknowledged by the Zambia Prisons Service [39]. The Zambian government also has an obligation to ensure its subjects have the right to enjoy the benefit of scientific progress and its applications [37,40].

Yet health conditions in Zambian prisons indisputably violate international prohibitions on cruel, inhuman or degrading treatment; and the medical care available, and support provided by international donors, is far from that available in the general population. Zambian law establishes minimum standards for medical care, and requires that the officer in charge of each prison maintain a properly secured hospital, clinic or sick bay within the prison [41]. A serious gap, however, exists between these legal requirements and practice, with little or no medical care available at most of Zambia's 86 prisons. Criminal justice system failures lead to extended pre-trial detention in violation of international law, and abuses of inmates' rights exacerbate overcrowding, poor conditions and inadequate medical care.

In addition to calling upon, and supporting, Zambia to respect its human rights obligations to prisoners, international donors should examine their own portfolios of health grant-making. International human rights law indicates that donors should honor the principles of non-discrimination and equality in their funding of such services as health [42,43]. While in the case of Zambia, donors have not specifically restricted their funding for prison health initiatives, the funding that they have chosen has failed to be applied equally and without discrimination to the vulnerable groups requiring it.

Certainly, public health strategies by national governments and international donors may use public health criteria to target services in ways that differ from a strictly equitable allocation of resources. However, given prisoners' higher rates of HIV and TB compared with the general population, and linkages between prison and non-prison populations facilitating disease transmission, including prisoners in Zambia's campaign against infectious disease can be seen as essential from both a human rights and public health perspective. High turnover in the prison population, coupled with the fact that prison officers and visitors travel frequently between prison settings and the general population, holds the potential for swift spread of disease both into and out of prison settings.

Recognition of the importance of protecting human rights in addressing HIV and TB vulnerability is often expressed by international agencies, and prisoners are a frequently cited "vulnerable" or "most at-risk" population [44-48]. Yet, despite this rhetorical commitment, in Zambia, little attention has been provided to the human rights of prisoners, both to equivalent medical care and to basic conditions of detention, leaving a population excluded from government guarantees of care, facilitating ongoing disease transmission and the development of multi-drug resistant pathogens. Although there is increasing global recognition that "good prison health is good public health" [49], in Zambia, prisoner health is of limited priority and negligible concern.

There were several limitations to our research. Prisoners in only six of 86 institutions were interviewed, and the recruitment of prisoners required the cooperation of prison officers. Because the prisons selected were participating in an ongoing HIV prevention programme run by a non-governmental organization (PRISCCA), and subject to visits by NGO staff, conditions may have been better in these prisons than in the 80 prisons not visited. Similarly, the selection of prisoners by prison officers likely biased the sample to healthy prisoners not currently in punishment cells, who were possibly more likely to portray prison staff and conditions in a positive light.

However, using mixed-method approaches and triangulating information from prisoners with in-depth interviews with prison officers and NGO and government representatives, as well as facility assessments, strengthened our confidence in our main findings. Even if our results suggest more positive conditions than those experienced by a more representative sample of Zambian prisoners, the findings identify serious human rights abuses and failures to provide healthcare that compel further investigation, monitoring and response by the Zambian government.

## Conclusions

This study presents the first published research conducted by international human rights monitors in Zambian prisons, and found that significant challenges exist in guaranteeing prisoners' human rights and adequate or equal access to health care, including HIV and TB prevention, testing and treatment. Greater resources are needed for prison-based medical services in Zambia, and accountability measures need to be developed to ensure that both the government and international donors ensure non-discrimination and equal access in the provision of health resources in the country.

Improving prison-based HIV and TB prevention and treatment, and general medical services, as well as eliminating the criminal justice system failures that contribute to overcrowding and extended pre-trial detention, are essential to protecting the human rights and health of inmates and the general population of Zambia.

## Competing interests

The authors declare that they have no competing interests.

## Authors' contributions

All authors conceived the study. KWT and JJA designed the research instruments and methodology, and KWT and GM led the field research. KWT and JJA drafted the manuscript, which GM and MC reviewed. All authors approved the final manuscript.
